# Persistent Microalbuminuria in Human Immunodeficiency Virus Infected Children in Kano, Nigeria

**DOI:** 10.1155/2014/567838

**Published:** 2014-03-03

**Authors:** Abdullahi Mudi, Bashir U. Alhaj, Fatimah Hassan-Hanga, Isah Adagiri Yahaya

**Affiliations:** ^1^Department of Paediatrics, Aminu Kano Teaching Hospital, PMB 3452, Kano, Nigeria; ^2^Department of Paediatrics, Aminu Kano Teaching Hospital/Bayero University, PMB 3452, Kano, Nigeria; ^3^Department of Chemical Pathology, Aminu Kano Teaching Hospital/Bayero University, PMB 3452, Kano, Nigeria

## Abstract

Microalbuminuria has been reported to be a precursor of HIV related renal disease, which if detected early and coupled with appropriate intervention may slow or retard the progress of the disease. One hundred and seventy-eight HIV infected children aged 15 years and below were recruited from the Paediatric Infectious Disease Clinic of Aminu Kano Teaching Hospital (AKTH), Kano, to determine the prevalence of persistent microalbuminuria using the albumin creatinine ratio (ACR). Early morning urine samples and spot urine samples were analyzed using a dipstick specific for microalbumin. Those who tested positive had their samples reanalyzed in the laboratory using immunometric assay and Jaffe reaction method for albumin and creatinine, respectively. Patients that had ACR of 30–300 mg/g were said to have microalbuminuria and had their urine samples retested after 6 to 8 weeks. Twelve children (6.7%) had persistent microalbuminuria and had a mean age of 7.5 ± 3.3 years, with a male to female ratio of 1 : 1. There was no significant relationship between the finding of microalbuminuria and age, sex, duration of infection, and the use of highly active antiretroviral therapy. Periodic screening for microalbuminuria using albumin specific dipstick should be considered for children with HIV infection.

## 1. Introduction

The human immunodeficiency virus (HIV) infection is a global pandemic, with cases reported from virtually every country. At the end of 2010, an estimated 34 million people were living with HIV globally, including 3.4 million children less than 15 years [1]. In 2010, an estimated 250 000 children less than 15 years died from AIDS-related causes [1]. In Nigeria, an estimated 3.6 percent of the population is living with HIV and AIDS [2]. Unlike in adults where more than 90% of HIV infections occur through sexual route, in children 95% of cases occur due to vertical transmission from their infected mothers [3].

The kidney is one of the many organs involved with the progression of HIV infection. Renal involvement in HIV infected children is a common finding [3] and HIV-associated nephropathy (HIVAN) is a type of kidney disease that occurs in patients who are infected with HIV [3]. Highly active antiretroviral therapy (HAART) has been found to be beneficial not only in long term patient survival but also slowing down renal involvement and rapid progression to end stage renal disease [3].

Microalbuminuria refers to albumin excretion above the normal range, but below the level of detection by dipstick [4]. Microalbuminuria is defined as urinary albumin excretion between 30 and 300 mg/day or in concentrations 20 to 200 *μ*g/min [4, 5]. Microalbuminuria is also defined as 30 to 300 mg albumin/g creatinine on a first morning urine specimen [4, 5].

Microalbuminuria has been studied in many diseases such as diabetic nephropathy, hypertension, obesity, HIV, and sickle cell disease [6]. Microalbuminuria develops from progressive, subclinical, structural, and functional changes in the kidney and it is useful as an early biomarker in the detection of kidney disease [6]. Microalbuminuria and proteinuria are believed to be precursors of HIV related renal disease; early detection with intervention may prevent end stage renal disease (ESRD) in HIV infected children [7].

Chronic kidney disease and end stage renal disease pose enormous problems to patients and physicians alike for reasons which, in no small part, have to do with the lack of resources needed for their management [8]. Treatment of ESRD relies on dialysis and transplantation, which are very expensive and often unavailable [8]. Transplantation is currently available in Nigeria in only a few hospitals and costs over 3 million naira, while dialysis can cost over 100 000 naira monthly [8]. Consequently, preventive measures offer the greatest hope in the management of patients with chronic kidney disease. Primary prevention (nephroprevention) is advocated for those subjects in the general population at risk of progressive renal failure [9–11].

Any cost effective, sensitive, and selective marker that allows patient identification as low- or high-risk would facilitate the choice of appropriate intervention, to provide optimal risk-benefit ratio. Such intervention can subsequently be integrated into a clinical decision-making protocol.

Screening for microalbuminuria, a simple and relatively inexpensive procedure may identify HIV infected patients with impaired renal function early and enable them to benefit from measures which may slow or halt the progression of disease [10]. This may reduce cost of care and possibly improve survival.

## 2. Methods

*Study Site.* The study was conducted in the Paediatric Infectious Disease Clinic (PIDC) of Aminu Kano Teaching Hospital (AKTH), Kano, where HIV infected children are seen and followed up.

*Study Design*. Study design was longitudinal, conducted from July 2012 to November 2012. 

*Study Population*. Study population was HIV infected children that presented newly or on followup at the PIDC. Fifteen years is the upper age limit for paediatric patients seen in the hospital. Children who satisfied the inclusion criteria were enrolled in the study. 

*Inclusion Criterion*. Inclusion criterion was all HIV infected children between the ages of 1 and 15 years at the time of enrolment who presented newly or on followup at the PIDC of AKTH during the study period. 

*Exclusion Criteria. *Exclusion criteria were as follows:

1. children with haematuria or proteinuria, evidenced by dipstick urinalysis of trace or greater,
2. children with diabetes mellitus, hypertension,
3. children with fever at presentation,
4. children with ongoing menstruation,
5. children with overt clinical signs and symptoms of renal disease or on followup at the paediatric nephrology clinic,
6. refusal of consent from parent(s) or accompanying caregiver(s).

*Sample Size*. Sample size was determined using the formula [12] *n* = *z* ^2^ *p*(1 − *p*)/*d* ^2^, where “*n*” is the desired sample size, “*z*” is the standard normal deviate corresponding to 95% confidence level = 1.96, “*p*” is the prevalence of microalbuminuria in HIV infected children: 12% (reported in Port Harcourt, Nigeria) [13], and “*d*” is the absolute sampling error that can be tolerated = 0.05. To allow for attrition, 10% of the sample size was added to the calculated minimum sample. The sample size used for the study was 178. 

*Ethical Approval*. Ethical approval for the study was obtained from the ethical committee of the hospital. 

*Informed Consent*. Informed consent was obtained from the parent(s) or the accompanying caregiver(s) of each child after explaining the nature and purpose of the study. Assent was obtained from the patients where applicable. 

*Sampling Method.* A stratified sampling technique was used to recruit children into the study. The PIDC register was obtained and the case/medical file of the patients attending the HIV clinic reviewed to determine age bracket distribution of 1–5 years, 6–10 years, and 11–15 years. A review of the records showed a proportionate distribution of children as 30%, 47%, and 23%, respectively. The desired sample size in each stratum was then calculated as 54, 83, and 41. Using systematic sampling technique, a sampling interval of 1 in 3 was obtained. The first child was selected at random and, subsequently, every third child who met the inclusion criteria was then recruited until the desired number was obtained for each stratum. 

*Data Collection.* During a routine clinic visit, patients who met the study criteria were recruited from the PIDC of AKTH. A proforma designed for the study was used. It was administered by the investigator to the parents/caregivers or where appropriate to the child. Physical examination was carried out with emphasis on general examination, anthropometry, and blood pressure measurement. 

*Sample Collection.* The procedure was explained to the parents/caregivers and to the patients if old enough to understand. All patients were provided with properly labelled universal bottles for collection of first batch samples, that is, first morning void collected at home and spot urine samples collected on presentation at the clinic. Parents or accompanying caregivers of urine continent patients were instructed on collection of midstream void and clean catch specimen for noncontinent patients. About five millilitres of urine was required in each specimen bottle. The urine specimens were delivered to the hospital within 2 hours of collection or refrigerated and transported as soon as possible. The specimens were then retrieved by the investigator for analysis. For those patients that tested positive for microalbuminuria from analysis of the first batch sample, a follow-up second batch sample (first morning void only) was collected after 6 weeks for analysis. The age groups 1–5 years and ≥6 years had equal prevalence of microalbuminuria of 3.37%, respectively (Table 5). 

*Laboratory Methods.* Macroscopic and dipstick examination of urine was carried out by the researcher following the manufacturer's instructions using dipstick specific for microalbumin and creatinine, Combostick 2AC. Microalbuminuria was defined as 30 to 300 mg albumin/g creatinine. Samples that tested positive for MA using the microalbumin and creatinine specific dipstick were subjected to more technical procedures including urine creatinine and microalbumin quantitative measurements in the laboratory using the U-Albumin immunometric assay method [14] and the Jaffe reaction method [15], respectively. This was conducted by the laboratory scientist in collaboration with the researcher and supervising chemical pathologist.

The findings of the clinical examination and laboratory investigation were communicated to the caregivers and patients. Patients with overt renal symptoms such as hypertension, proteinuria, or haematuria were referred to the paediatric nephrologist for further evaluation and management. 

*Data Analysis.* Statistical analysis was conducted using the statistical package for the social sciences (SPSS) version 16.0. Data was presented using tables. Quantitative variables were summarized using the mean and median, while qualitative variables were summarized using percentages. Comparison of means was done using the Student *t*-test and Chi square test was used to test association between microalbuminuria and patient characteristics.

## 3. Results

### 3.1. General Characteristics of the Study Participants

One hundred and seventy-eight children were recruited for the study and a response rate of 100% was recorded. The mean age of the study participants was 7.5 ± 3.3 years. The youngest participant was 1-year old. One hundred and three (57.9%) participants were males while 75 (42.1%) were females, giving a male to female ratio of 1.4 : 1 (Table 1).

The mean weight of the study participants was 21.3 ± 7.6 kg with a range of 9 to 52 kg (Table 2). The mean height was 115.8 ± 20.1 cm (Table 2). All the participants had normal blood pressure with a mean systolic and diastolic blood pressure of 79.7 ± 12.8 mm/Hg and 52.0 ± 9.7 mm/Hg, respectively (Table 2).

The mean duration of infection in the study participants was 47.9 ± 29.1 months as indicated in Table 2. The majority of the study participants, 144 (80.9%), were classified as having WHO stage 1 disease, with only 2 (1.1%) participants having stage 3 disease and none with stage 4 disease (Table 1). Also, the majority of the study participants, 157 (88.2%), were on antiretroviral therapy (ART), while 21 of them (11.8%) were yet to HAART (Table 1). The mean CD4 cell count was 918.9 ± 487.4 cells/*μ*L. For those on ART, their mean duration of treatment was 39.5 ± 28.0 months (Table 2).

The study participants were also classified based on their immunological status using their absolute CD4 count. This classification was guided by the CDC immunologic classification for various age groups in HIV infected children [12]. The majority of the study participants, 127 (71.4%), had no evidence of suppression, whereas only 6 (3.4%) of them had severe suppression based on the CDC immunological classification (Table 3).

### 3.2. Prevalence and Sex Distribution of Microalbuminuria in the Study Participants

Twelve participants had persistent microalbuminuria, giving a prevalence of 6.74%. Sex specific prevalence of 3.37% was found in males and females, respectively. There was no statistically significant difference between gender and the presence of persistent microalbuminuria (Table 4).

### 3.3. Microalbuminuria in Early Morning and Spot Samples

All participants provided early morning and spot urine samples during the first batch of samples collection. Twelve (6.7%) tested positive for microalbuminuria using the EMS and 11 (6.2%) tested positive for microalbuminuria using the SUS. That is, only one (8.3%) out of the 12 that tested positive for microalbuminuria using EMS tested negative using the SUS. There was no significant statistical difference in the use of EMS or SUS in screening for microalbuminuria, *χ* ^2^ = 0.05, *P* = 0.83.

The mean values of microalbuminuria in early morning sample (EMS) and spot urine samples (SUS) were 65.8 ± 30.9 and 56.7 ± 30.8 mg/g, respectively. There was no statistical difference between the two mean values, *P* = 0.56. There was also no significant difference between the mean values of microalbuminuria of the two early morning samples (taken at least 6 weeks apart); *P* value was 0.148 (Table 6).

### 3.4. Microalbuminuria and Duration of Infection

The mean duration of HIV infection in participants with and without microalbuminuria was 42.8 ± 32.8 and 48.3 ± 28.8 months, respectively. There was no statistical difference between the mean duration of HIV infection between children with microalbuminuria and those without microalbuminuria, *t* = 0.63 with *P* value of 0.528 (Table 7).

### 3.5. Microalbuminuria and Use of Highly Active Antiretroviral Therapy

Eleven (91.7%) out of the 12 participants with microalbuminuria were on HAART, while only 1 (8.3%) of the participants with microalbuminuria was yet to commence HAART. There was no significant difference between the use of HAART and the finding of microalbuminuria, *P* = 1.0 (Table 8).

### 3.6. Microalbuminuria and Duration of HIV Treatment

The mean duration of HIV treatment in participants with and without microalbuminuria was 38.3 ± 35.3 and 39.5 ± 27.6 months, respectively. There was no statistical difference between the mean duration of treatment and the presence of microalbuminuria, *t* = 0.14 with *P* of 0.887 (Table 8).

## 4. Discussion

The prevalence of microalbuminuria in this study was found to be 6.7%. This finding is lower than the reported prevalence of 10–33% reported from Port Harcourt, Nigeria, Africa, India, and the United States [13, 16–20]. It is, however, higher than 0% reported from Enugu, Nigeria [21], and France [22], respectively.

Comparison of this study with previous ones was made difficult by various factors which include differing cut-off values for the definition of microalbuminuria, differing assay techniques, and widely varying sample sizes, in addition to widely varying study population and selection criteria. The finding of a lower prevalence in the index study when compared to others [13, 16–19] that reported higher prevalence may be explained by the various factors mentioned above. For example, the majority of the studies with a higher prevalence [13, 16–19] than this study recruited HAART naïve patients for their studies, whereas in this study both HAART naïve patients and patients on treatment with HAART were enrolled.

The laboratory methods used in this study were similar to those used in the Tanzanian study [19]. A dipstick specific for microalbumin and creatinine expressed as albumin creatinine ratio (ACR) was used on spot urine samples in both studies. However, the Tanzanian study had a larger sample size of 240 participants and the authors reported a higher prevalence of microalbuminuria of 20.4% and also reported that a lower CD4 count (<25%) was a risk factor for microalbuminuria (*P* < 0.01). The authors were, however, silent on the use of HAART. In this study, the majority (88.8%) of the patients were on HAART and had no evidence of immunological suppression (71.4%). This may account for the lower prevalence observed in this study.

The wide variation in reported prevalence may not be solely explained by differences in methodology, since studies with relatively similar methodologies also reported varying prevalences. The role of other factors such as genetics and environment may need to be considered. Patients with factors like obesity, hypertension, puberty, family history of hypertension and diabetes mellitus, and sickle cell anaemia have been observed to have higher prevalence of microalbuminuria when compared to those without them as reported by Okpere et al. [23] and Ibadin et al. [24].

Genetic factors are infrequently considered in published studies. Though hypertension and diabetes mellitus were considered as part of the exclusion criteria in this study, the family history of these conditions was not assessed for this study's participants.

We found no association of age and gender of the subjects with the prevalence of microalbuminuria. This finding is similar to that of the study conducted in India (2012) by Shah et al. [16] where it was observed that microalbuminuria in HIV infected children was not related to age and gender. Previous studies in Tanzania [19] and Port Harcourt [13], however, observed a higher prevalence of microalbuminuria in older children (>10 years) who also had a longer duration of infection and advanced disease. On the other hand, a study conducted in Uganda [17] reported that the majority of the children who had microalbuminuria were less than 5 years and were not on HAART. These findings revealed that even though microalbuminuria may be related with age, other clinical parameters such as duration of infection, use of HAART, and immunologic status may also influence the finding of microalbuminuria. In this study, no significant relationship was observed between gender and microalbuminuria which has been corroborated in many of the previous studies [13, 16–19].

In this study, duration of infection, use of HAART, and duration of treatment were analyzed for an association with microalbuminuria and no significant statistical difference was found between these clinical factors and microalbuminuria. This observation is similar to the findings by Shah et al. [16], who reported that the manifestation of microalbuminuria does not always correlate with duration and severity of HIV infection. On the contrary, most of the studies discussed earlier [13, 17–20] indicated that the presence of microalbuminuria was more prevalent in patients with moderate to severe immunosuppression or advanced disease who were also HAART naïve. Eke et al. [13] in the Port-Harcourt study reported a prevalence of 12%, where the majority of patients that had microalbuminuria (83.3%) had clinical AIDS and severe immunosuppression. Mistry [18] in a South African study also reported that patients with microalbuminuria were moderately immunosuppressed and had WHO clinical stages 2 and 3 disease.

All the studies [13, 16–18] that recruited only HAART naïve subjects had a higher prevalence than that observed in this study, while those studies [21, 22] that reported zero prevalence recruited only patients on HAART who had no advanced disease. This can be explained by the fact that the use of HAART slows or even reverses the progression of HIV related kidney disease. The study in Enugu [21] that reported zero prevalence recruited patients on HAART and most had early disease with no evidence of immunosuppression. The conclusion reached was that microalbuminuria occurs mainly in children who were not on HARRT.

There was no significant statistical difference between the findings of microalbuminuria in the early morning urine samples (EMS) and spot urine samples (SUS) from this study. Where albumin creatinine ratio (ACR) is evaluated, SUS may be considered to screen for microalbuminuria. However, there is a need for larger studies to strengthen the findings of this study.

The small sample size used for this study may have affected the results and probably the reason for the lack of association of microalbuminuria with patient characteristics identified in this study.

## 5. Conclusion

Microalbuminuria is believed to be a precursor of HIV related renal disease. Screening for microalbuminuria may identify HIV infected patients with impaired renal function early and enable them to benefit from measures which may slow or halt the progression of disease. In this study, microalbuminuria was observed in some of the children with HIV infection seen at AKTH and there was no significant relationship between the finding of microalbuminuria and age, gender, duration of infection, use of HAART, and duration of treatment. There was also no significant difference between the use of EMS and SUS in screening for microalbuminuria in HIV infected children seen in AKTH. In an attempt to improve patient compliance and acceptability, SUS may be considered in place of EMS to screen for microalbuminuria. Periodic screening for microalbuminuria using albumin specific dipstick, a simple and relatively inexpensive procedure, should be considered for children with HIV infection. Furthermore there is the need to conduct further studies to establish the natural course of microalbuminuria in children with HIV infection.

## Figures and Tables

**Table 1:** General characteristics of the study participants.

General characteristics *N* = 178	
	*n* (%)
Age group (years)	
1–5	54 (30.3)
6–10	83 (46.6)
11–15	41 (23.1)
Sex	
Male	103 (57.9)
Female	75 (42.1)
WHO classification of HIV/AIDS	
Stage 1	144 (80.9)
Stage 2	32 (18.0)
Stage 3	2 (1.1)
Stage 4	0 (0)
ART use	
Yes	157 (88.2)
No	21 (11.8)

WHO: World Health Organisation.

**Table 2:** Descriptive statistics of the study participants.

	Mean ± SD	Range
Age (years)	7.5 ± 3.3	1–15
Weight (kg)	21.3 ± 7.6	9–52
Height (cm)	115.8 ± 20.1	70–185
SBP (mm/Hg)	79.7 ± 12.8	50–110
DBP (mm/Hg)	52.0 ± 9.7	30–80
Duration of infection (months)	47.9 ± 29.1	2–150
CD4 count (cells/*μ*L)	918.9 ± 487.4	103–2913
Duration of treatment (months)	39.5 ± 28.0	0–120

SBP: systolic blood pressure; DBP: diastolic blood pressure.

**Table 3:** Immunologic classification of the study participants based on the CDC immunologic classification.

Age group (years)	No evidence of suppression *n* (%)	Moderate suppression *n* (%)	Severe suppression *n* (%)	Total
1–5	37 (68.5)	16 (29.6)	1 (1.9)	54 (100)
≥6	90 (72.6)	29 (23.4)	5 (4.0)	124 (100)
Total	**127 (71.4)**	**45 (25.3)**	**6 (3.4)**	**178 (100)**

**Table 4:** Sex distribution of study participants with and without microalbuminuria.

Sex	Microalbuminuria		Total *n* (%)
	Positive *n* (%)	Negative *n* (%)	
Male	6 (5.8)	97 (94.2)	103 (100)
Female	6 (8.0)	69 (92.0)	75 (100)
Total	**12 (6.7)**	**166 (93.3)**	**178 (100)**

*χ* ^2^ = 0.33, *P* = 0.56, df = 1.

**Table 5:** Age groups of participants with and without microalbuminuria.

Age group (years)	Microalbuminuria		Total *n* (%)
	Positive *n* (%)	Negative *n* (%)	
1–5	6 (11.1)	48 (88.9)	54 (100)
≥6	6 (4.8)	118 (95.2)	124 (100)
Total	**12 (6.7)**	**166 (93.3)**	**178 (100)**

Fisher's exact, *P* = 0.19.

**Table 6:** Microalbuminuria in early morning and spot urine samples.

Sample	Microalbuminuria		Total *n* (%)
	Positive *n* (%)	Negative *n* (%)	
EMS	12 (6.7)	166 (93.3)	178 (100)
SUS	11 (6.2)	167 (93.8)	178 (100)

*χ* ^2^ = 0.05, *P* = 0.83, df = 1.

**Table 7:** Mean of various variables in participants with and without microalbuminuria.

Variables	Microalbuminuria		*t* value	*P* value
	Positive (*n* = 12)	Negative (*n* = 166)		
	Mean ± SD	Mean ± SD		
Age (years)	6.8 ± 3.4	7.6 ± 3.3	0.81	0.419
CD4 count (cell/*μ*L)	1000.0 ± 523.3	913.0 ± 485.8	0.60	0.552
Duration of infection (months)	42.8 ± 32.8	48.3 ± 28.8	0.63	0.528
Duration of treatment (months)	38.3 ± 35.3	39.5 ± 27.6	0.14	0.887

**Table 8:** Microalbuminuria and use of highly active antiretroviral therapy.

	Microalbuminuria		Total
	Positive *n* = 12 (%)	Negative *n* = 166 (%)	
HAART			
Yes	11 (7.0)	147 (93.0)	158 (100)
No	1 (5.0)	19 (95.0)	20 (100)
Total	**12 (6.7)**	**166 (93.3)**	**178 (100)**

Fisher's exact, *P* = 1.00.
